# Evaluating the Efficacy of the Family Check-Up Online to Improve Parent Mental Health and Family Functioning in Response to the COVID-19 Pandemic: A Randomized Clinical Trial

**DOI:** 10.1007/s10935-023-00727-1

**Published:** 2023-03-16

**Authors:** Arin M. Connell, Elizabeth A. Stormshak

**Affiliations:** 1 Department of Psychological Sciences, Case Western Reserve University, 44106 Cleveland, OH USA; 2 University of Oregon, Eugene, USA

**Keywords:** Family Intervention, Parenting, COVID-19 pandemic, Remote intervention

## Abstract

This study evaluated the effects of an enhanced version of the Family Check-Up Online (FCU-O), adapted to address parent and family functioning in response to the COVID-19 pandemic. In order to increase accessibility, the FCU-O was delivered as a web-based application coupled with online coaching support, a service delivery model that is consistent with pandemic-related limitations for in-person intervention, as well as the limited staffing and resources available in many schools and health care settings despite the increased need for mental health services driven by the pandemic. This registered clinical trial (*blinded*) tested the effects of the intervention on parental mental health, parenting behaviors, and family functioning from pre-treatment to 2-month follow-up. Families were randomly assigned to receive the FCU-O (N = 74) or to a wait-list control condition (N = 87). Random assignment to the FCU-O was associated with significant improvements in parental well-being, including reduced anxiety, depression, and perceived stress. Further, the FCU-O predicted significant improvements in adaptive parenting skills (e.g. less negative/coercive parenting, greater proactive parenting), and enhancements in family-relational functioning (e.g. improved coparenting). Effect sizes were small to moderate in magnitude (partial eta squared values between 0.03 and 0.11). The results indicate that online delivery of a family-centered intervention may represent a promising approach for addressing pandemic-related impacts on parent and family functioning.

## Introduction

The COVID-19 pandemic created significant disruptions for families. In the US, schools abruptly transitioned to remote instruction in March 2020, leading to substantial challenges for parents, who had to navigate home schooling with little preparation for doing so effectively. Other adverse effects of the pandemic on parents included economic stressors (e.g. changes in employment status or functioning), mental health difficulties (including depression and anxiety), alterations in social/familial functioning (including parenting and risk for domestic violence), and negotiating safety measures (e.g. social distancing, stay-at-home orders, mask-wearing; WHO, 2020). Children and adolescents were also heavily impacted by the pandemic, and increases in youth emotional, behavioral, and academic difficulties (e.g. Engzell et al., 2021; Thorisdottir et al., 2021; Waller et al., 2021) represented another significant stressor for parents. Such effects are likely to persist far beyond the acute phase of the pandemic (e.g. Kaffenberger, 2021), and many school systems have reported increased need for services to to address elevated rates of social, emotional, and academic difficulties among youth following the return to in-person schooling (Karaman et al., 2021).

### Web-based Behavioral Interventions

The COVID-19 pandemic has highlighted the urgent need for effective family-focused interventions that can be delivered remotely to support families during times of overwhelming disruption (Stormshak et al., 2021). As internet usage increases, so does the potential reach of online or telehealth interventions. Although researchers have successfully adapted evidence-based parenting curriculums to web-delivery (Enebrink et al., 2012; Feil et al., 2008; Sanders et al., 2012), this work has primarily focused on younger children, with little attention to school-age children or delivery in community settings. Moreover, most were developed for computer-access (Breitenstein et al., 2014), although smartphone capability is increasingly important, particularly for low SES families who are more likely to own a mobile phone than a computer (e.g. Shields et al., 2018). Despite such limitations, a review of the literature suggests that online family-centered interventions have promising acceptability to users, which may be enhanced for at-risk families if coupled with a coach or virtual support (Hall & Bierman, 2015). Further, a recent meta-analysis of remotely-delivered family-focused interventions across childhood and adolescence indicated that they can be equally effective as in-person treatments, at least for some outcomes, including improvements in maternal depression and youth conduct problems (McLean et al., 2021). Such findings support the use of web-based or app-delivered interventions (i.e. “eHealth” interventions) to improve mental health.

### Family Check-Up

Given the scale of pandemic impacts for families with school-aged children, the identification of effective interventions targeting core mechanisms of change that can be brought to scale rapidly and with fidelity represent critical public health goals. One promising intervention is the Family Check-Up (FCU), which was originally developed as an in-person treatment program, employing motivational interviewing strategies to engage families in behavioral change with the long-term goal of reducing mental health and behavior problems (Dishion & Stormshak, 2007). FCU development was guided by an ecological model emphasizing that contextual stressors such as poverty and stress directly limit parents’ ability to respond to their child with effective parenting. The goal of the FCU is to support positive, proactive parenting strategies, in order to disrupt the developmental cascades from contextual stressors to youth emotional and behavioral problems. In its original in-person form, the FCU includes an up-front 3-session family assessment that leads to a feedback session in which the therapist summarizes assessment results using motivational interviewing strategies, and explores intervention services that support family management practices. The outcome of the assessment is the identification of intervention options, including family-based intervention tailored to the individual goals of the family. In multiple randomized trials with ethnically and socioeconomically diverse youth, the FCU has been shown to reduce parental depression (e.g. Shaw et al., 2009), and improve adaptive parenting and family functioning (e.g. Brennan et al., 2013; Van Ryzin et al., 2012). In turn, such changes are associated with long-term benefits for youth, including improvements in youth self-regulation, academic performance, depression, and antisocial behavior across childhood and adolescence (e.g. Fosco et al., 2013; Stormshak et al., 2009; 2010; Connell et al., 2021).

### Family Check-Up Online

In order to enhance accessibility, the FCU was recently adapted for online delivery (Stormshak et al., 2019). The FCU Online (FCU-O) was developed with feedback from community partners as an eHealth approach to reduce problem behavior in early adolescence (Danaher et al., 2018; Stormshak et al., 2019). It includes online materials incorporating parent-assessments with feedback, and modules on effective parenting that are adapted from the Everyday Parenting Curriculum (Dishion et al., 2012), along with supplemental telehealth coaching that includes at least three phone or video-conferencing sessions to support parents in making behavioral change (Danaher et al., 2018; Stormshak et al., 2019).

A prior randomized trial with parents of 322 middle-school aged youth compared an assessment-only control condition with two versions of the FCU-O, one including only the online materials, and a second including online materials plus coaching support (Stormshak et al., 2019). The FCU-O with coaching support improved parents’ self-efficacy and child emotional problems at three months post-test, with higher behavioral risk associated with greater improvements (Stormshak et al., 2019). For children with higher levels of behavior problems, the FCU-O also produced effects on effortful control and parenting confidence, key mechanisms of change.

### Current Study

In response to the COVID-19 pandemic, the FCU-O was adapted to include new intervention material developed to enhance the FCU-O’s focus on supporting adaptive parenting and coping with stress (including stress associated with the pandemic). Additionally, intervention materials were modified for delivery via a smartphone app rather than via the web-based platform used in the prior trial (Stormshak et al., 2019). The brief and tailored approach to intervention makes the FCU-O with telehealth coaching ideally suited for community mental health agencies that want a brief, cost-effective, targeted parent support model for youth in need of supports that can be delivered remotely. The current study examined two-month outcomes in an RCT comparing the adapted FCU-O with a wait-list control condition, for families of youth aged 10–14 years recruited from community health agencies, schools, and pediatric settings predominantly from urban areas of the Pacific Northwest.

In line with prior research on both the FCU and the FCU-O, we expected the adapted version of the FCU-O would predict improvements in several domains. First, in line with prior research documenting FCU effects on reductions in parental depression (e.g. Seidman et al., 2023), improvements in parenting confidence (Stormshak et al., 2019), improvements in self-regulatory abilities in youth (e.g. Fosco et al., 2013; Chang et al., 2014), we predicted that the FCU-O would be associated with improvements in parental mental health (including anxiety and depression), emotion regulation, and perceptions of stress. Second, we expected that the FCU-O would predict improvements in effective parenting skills (including greater engagement in positive, proactive parentint, parental monitoring, and limit-setting, as well as reductions in parental coercive/hostile parenting). Third, we predicted that the FCU would predict improvements in family relational functioning (including reductions in family conflict and improvements in family togetherness and co-parenting), in line with the results of prior research (e.g. Brennan et al., 2013; Dishion, Nelson & Kavanagh, 2003). We also examined moderation of intervention effects by youth gender and race/ethnicity. Prior research with the FCU has shown few differences in intervention effects on outcomes by gender or race/ethnicity, and so moderation analyses were exploratory. All hypotheses were pre-registered, with improvements in parent functioning and parenting skills considered primary outcomes, and improvements in family relational functioning considered secondary outcomes.

## Methods

### Participants

Participants were primary caregivers (N = 161) of children aged 10 to 14 years. Primary caregivers were predominantly female (n = 153; 95%), with an average age of 42.77 years (SD = 6.65). Parents self-identified as follows: European American (84.6%), Latinx (20.4%), multiracial (11.7%), Asian (1.9%), and African American (0.6%). Most parents (66.7%) had at least a 4-year college degree, and a median family income between $80,000 to $89,000, although 14.9% (n = 24) had annual incomes below the poverty level relative to family size. Parents were married/cohabiting in 83.2% (n = 134) of families. Children were approximately balanced by gender (50.9% Female).

### Procedures

Study procedures were approved by the University of Oregon IRB. Families were recruited using flyers distributed through middle schools, community agencies (e.g. Boys and Girls Clubs), pediatric settings (e.g. pediatricians offices), and through a statewide mailing to families of middle-school students providing COVID-testing kits. The flyer provided information to parents about the study and directed them to the study website, which provided further information about the project, and asked families to provide contact information to discuss participation. The target sample size was N = 160. During a screening phone call, research staff provided details regarding the study, and parents were asked complete brief screening measures for study eligibility. To be eligible, parents had to indicate that they were the legal guardian of a child aged 10 to 14 years, and that they had a smartphone with text-messaging capability and email access. Parents also had to endorse significant depression on the Patient Health Questionaire-2 (PHQ-2; Lowe, Krenke, & Grafe, 2005), and/or significant stress associated with the pandemic, using the 4 item version of the Perceived Stress Scale (PSS; Cohen, Kamarck, & Mermelstein, 1994). Families were excluded if they failed to meet the above inclusion criteria, if parents indicated that their child was not capable of completing a survey without parental help, if parents were not fluent in spoken and written English or Spanish, or if parents did not have a smartphone capable of accessing the app.

**Fig. 1:**
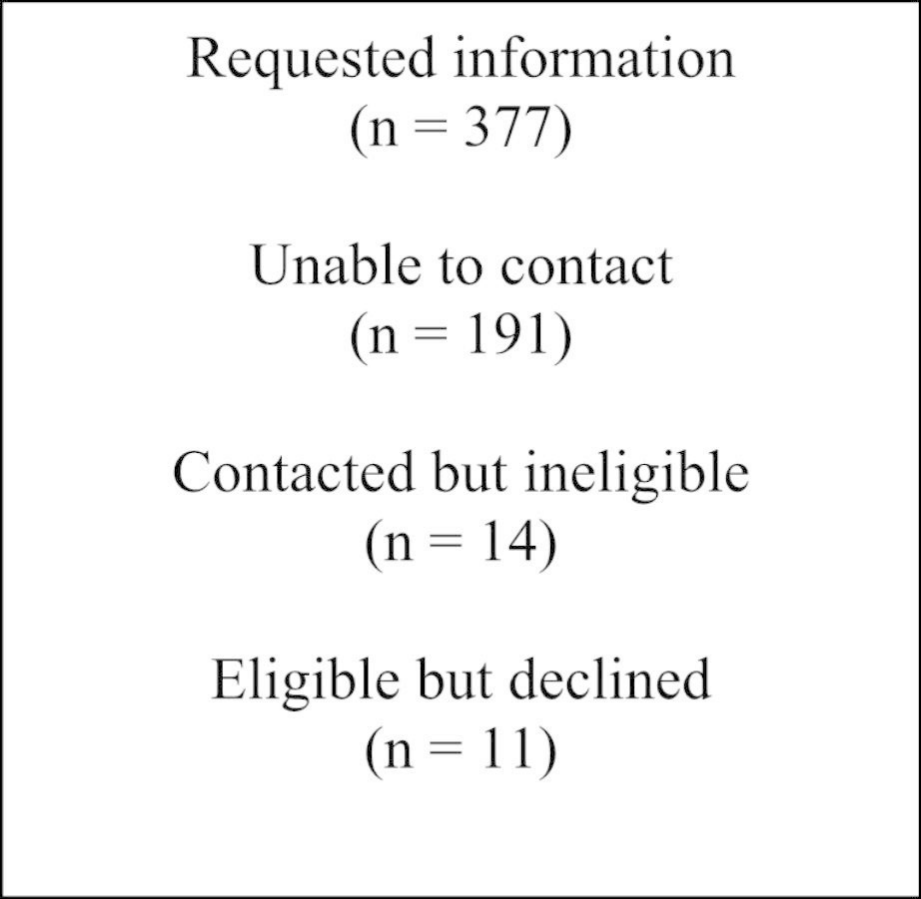
CONSORT Diagram

As shown in Figs. 1 and 377 families provided contact information, in order to discuss participation with study staff. Of these, study staff were unable to contact 191 families despite multiple efforts. The remaining 186 families were contacted, and responded to screening measures. Of the contacted families, 161 families (87% of contacted families) enrolled in the trial. Of the remaining families, 14 families did not meet eligibility criteria for participation, and 11 families (5.9% of contacted families) ultimately declined participation (4 indicated they were too busy, 1 wanted in-person intervention services, and the remainder indicated family concerns regarding participation). Family members who agreed to participate provided active consent, and families were block-randomized by child gender to treatment versus control conditions, by a research coordinator, to ensure balance across treatment arms.

### FCU-O Intervention Protocol

The FCU-O was adapted from the original online model (Stormshak et al., 2019) in multiple ways, in order to improve accessibility and support family functioning in relation to significant stressors, such as those associated with the COVID-19 pandemic. First, curriculum content was reduced to be accessible by smartphone. Key content and skill development was retained and summarized in brief, activity-based modules to elicit parent-engagement and skill development. A new module labeled “Healthy Behaviors for Stressful Times” was created, focused on supporting families in the development of healthy routines and structures for reducing anxiety and depression in response to stressful experiences such as the pandemic, including physical exercise, family activities, and academic support. Of note, intervention materials in this module were framed as supporting adaptive coping in response to stressful experiences, in general (rather than exclusively in response to the pandemic), and can be applied broadly to a range of circumstances, in order to enhance the broader utility of the app beyond the COVID-19 pandemic. This module was created in an iterative process, including feedback from focus groups with parents and community providers, who reviewed preliminary versions of the app-based materials and provided feedback regarding acceptability and useability. In addition to the new module, four other modules provided content consistent with prior FCU research including positive parenting, rules and consequences, support for school success, and communication. Modules were presented to families in a consistent order (“Healthy Behaviors for Stressful Times,” “Positive Parenting,” “Rules and Consequences,” “Support for School Success,” and “Communication”). However, there were no compulsory modules that parents had to engage with. Families received feedback from the app based upon the completion of in-app assessments, that highlighted some sections of modules as particularly relevant for areas of parenting or family functioning for which challenges were noted.

The FCU-O also includes remote meetings with parent coaches. Coaches were masters-level clinicians trained to fidelity using the COACH rating system (Smith et al., 2013). Coaches were randomly assigned to families after the baseline assessment. Coaches contacted families for an initial meeting to facilitate use of the FCU-O, set goals, and provide a framework for coaching. Subsequently, coaches monitored family progress through the app, and contacted families to schedule a coaching session after the completion of each module. Families could also contact coaches using the app to request coaching sessions. At all contacts, motivational interview was used to engage families in a process of change, connect with families around goal setting, and support families in behavioral skill development. Fidelity to the model was evaluated at multiple points for each therapist over the course of the intervention.

The FCU-O app and coaching sessions were offered in both English and Spanish, as were all assessment materials. Families could access the app and coaching sessions throughout the entire trial, although most app-visits (86%) occurred within the first two-months. Parents and youth completed baseline assessments. Parents also completed 2-month follow-up assessments.

### Wait-List Control Condition

During the intervention trial, the wait-list control condition completed assessments on schedule, and after the trial they received the same FCU-O content as the intervention group.

#### Measures

Demographic Characteristics and COVID-19 effects. Parents completed demographic questions for all family members, including gender, age, race/ethnicity, and indices of Socioeconomic Status. For child gender, response options were male, female, and other, and parents selecting “other” were asked to describe their child’s gender in a free-response field. For analyses, a two-category variable was used to reflect female gender (0 = male or any non-female “other” response; 1 = any female response). Parents also reported on challenges experienced during the pandemic (e.g. job-loss or changes, contracting COVID-19), and experiences with remote learning.

Perceived Stress Scale (PSS; Cohen, Kamarck, & Mermelstein, 1994). The PSS is a well-validated (Cohen et al., 1994) and widely used measure that includes 14 items scaled from 0 (never) to 4 (fairly often), with higher scores reflecting greater past-month stress (alpha at baseline = 0.90). For screening purposes, a 4-item version of the PSS was used, with scores of 2 or above on any item (sometimes) used to define eligibility.

Difficulties in Emotion Regulation Strategies (DERS; Gratz & Roemer, 2004). The “Impulse Control Difficulties” subscale includes 6 items reflecting difficulty maintaining self-control when upset (alpha at baseline = 0.76). The “Limited Access to Emotion Regulation Strategies” subscale has 8 items reflecting difficulty generating strategies to cope when upset (alpha at baseline = 0.86). The DERS is well-validated, and has been used extensively with adolescents and adults (see Kaufman et al., 2016).

GAD-2 (Kroenke, Spitzer, Williams, Monahan, & Lowe, 2007). This well-validated 2-item measure assesses core symptoms of Generalized Anxiety Disorder in the past 2-weeks, with responses reflecting the frequency with which symptoms were experienced (from 0 “not at all” to 3 “nearly every day”). Scores of 3 or above reflect significant symptoms (Kroenke et al., 2007).

Patient Health Questionnaire-2 and − 9 (PHQ-2; Kroenke, Spitzer, & Williams, 2003; PHQ-9; Kroenke, Spitzer, & Williams, 2001). The PHQ-2 has been validated for use as a depression screener. Items reflect the frequency with which symptoms of depressed mood or anhedonia were experienced in the past two-weeks, with responses ranging from 0 (not at all) to 3 (nearly every day). Scores of 1 or above (experiencing symptoms for several days) were used for screening eligibility purposes, a cutoff with high sensitivity for screening in the population (Kroenke et al., 2003). Baseline and 2-month follow-up assessments used the PHQ-9, a 9-item measure assessing depression severity in the past 2-weeks with the same response-scale as the PHQ-2 (alpha at baseline = 0.86). Scores of 5–9 reflect mild depression, and 10 or above reflect at least moderate severity (Kroenke et al., 2001).

Parenting Young Children (PARYC; McEachern et al., 2012). Parents completed an adolescent adaptation of the PARYC. Validity evidence includes concurrent and predictive associations with established measures of parenting and youth emotional and behavior problems (e.g. McEachern et al., 2012). The measure includes the following subscales: (1) positive parenting, 7-items reflecting the use of praise and positive reinforcement (alpha at baseline = 0.79); (2) proactive parenting, 7-items reflecting planning ahead to minimize problems with youth (e.g. breaking tasks into small steps; alpha at baseline = 0.79); (3) limit-setting, reflecting setting rules, expectations, and consequences (alpha at baseline = 0.76); (4) negative/coercive parenting, 7-items reflecting parental coercion and criticism of youth (alpha at baseline = 0.70); (5) monitoring school activities, 5-items reflecting parental knowledge of youth behavior while at school (alpha at baseline = 0.71) (6) monitoring routines, 8-items reflecting parental knowledge of youth activities when away from parents (alpha at baseline = 0.74).

Parents also completed three family-relationship subscales including: (1) family togetherness, 3-items reflecting family cohesion (alpha at baseline = 0.84), (2) family conflict, 4-items reflecting conflict frequency (alpha at baseline = 0.68), and (3) Supportive co-parenting, 6-items (completed by parents in a relationship; alpha at baseline = 0.82).

### Data Analysis

Preliminary analyses included descriptive analyses regarding COVID-19 impacts, as well parental engagement with components of the FCU-O. Hypothesis-testing analyses of intervention effects were tested with regression-based ANCOVAs, with Time 2 outcomes regressed on a binary treatment-status variable (0 = wait-list control; 1 = FCU-O), while controlling for Time 1 scores on the outcome variable. This approach provides powerful unbiased estimates of intervention effects (O’Connell et al., 2017). Hypotheses were pre-registered with ClinicalTrials.gov, and separate analyses were conducted for each outcome variable. A priori power analyses indicated that the study has 80% power for a Minimum Detectable Effect of Cohen’s d ≥ 0.24 (a small to moderate effect), assuming normally distributed outcomes and a dichotomous treatment indicator. These assumptions are reasonable for most outcome variables examined in the current analyses, although power may be somewhat lower for three variables that exhibited significant skew at baseline, including Impulse Control Difficulties (skew = 1.07), Access to Strategies Difficulties (skew = 1.84), and Monitoring Family Routines (skew = -1.22). Effect size estimates are presented as Cohen’s d, with values of 0.20 = small; 0.50 = medium; 0.80 = large (Cohen, 1988).

We also examined child gender (0 = male/other; 1 = female) and racial/ethnic minority status (0 = European American; 1 = racial/ethnic minority) as moderators of intervention effects. Moderation models included main effects of treatment assignment, moderator, and T1 scores for outcome, and the treatment by moderator interaction. A priori power analyses indicated that the study had 80% power for a Minimum Detectable Effect Size Difference across subgroups of d ≥ 0.48, a medium difference in effects.

## Results

### Preliminary Analyses

Demographics and missing data. Demographics factors are shown in Table 1. There were no statistically significant differences in demographic factors across intervention and control conditions. Of 161 families completing baseline assessments, 140 (87%) also completed two-month follow-up assessments. We examined Little’s (1988) Missing Completely at Random (MCAR) test, including all baseline and two-month follow-up variables, child gender, race/ethnicity, and intervention assignment. Little’s MCAR test was not significant (χ2 [811] = 803.31, p = .51), indicating that the data were consistent with MCAR assumptions.

**Table 1:** Sample demographics by intervention assignment

	Wait-list (n = 87)			Intervention (n = 74)	
	n	%		n	%
Parent’s gender (female)	83	95.4		70	94.6
Child’s gender (female)	46	52.9		36	48.6
Parent’s race/ethnicity					
White/Caucasian	74	85.1		62	83.8
Black/African American	0	0		1	1.4
Asian	2	2		1	1.4
Multiracial	9	9		10	13.5
Unknown	2	2		0	0
Parent Hispanic/Latinx	18	20.7		14	18.9
Parent’s education level					
Less than high school degree	3	3.4		2	2.8
High school degree or GED	7	8		6	8.1
Partial college	9	10.3		10	13.5
2-year associate’s degree	6	6.9		10	13.5
4-year college degree	33	37.9		24	32.4
Graduate/professional training	29	33.3		22	29.7
Child’s grade					
4th	1	1.1		2	2.7
5th	6	6.9		3	4.1
6th	42	48.3		26	35.1
7th	20	23		26	35.1
8th	18	20.7		15	20.3
9th	0	0		2	2.7
Primary caregiver single	18	20.7		10	13.5
Family below poverty-level	13	14.9		11	14.9
Primary caregiver unemployed	6	6.9		5	6.8
Number of children	Mean = 2.10 (range = 1 to 5)			Mean = 2.14 (range = 1 to 5)	

COVID-19 impacts. Oregon implemented remote learning due to the pandemic, on March 19, 2020. By April 19, 2021, Oregon schools were required to return to hybrid or fully in-person instruction. Recruitment for the current trial began in December, 2021 and continued through the end of March 2022. By the start of the current trial, most youth were receiving fully in-person instruction (n = 140; 87.5%). During the remote-instruction phase, parents reported that their children spent an average of 3–4 h per day on remote-learning activities, while parents spent an average of 1–2 h per day helping with remote learning. Most parents (n = 110; 68.8%) reported that remote instruction caused “a lot” of stress for their family (a score of 4 on a scale from 1 = “none” to 4 = “a lot”). Many families (n = 72; 44.7%) had an immediate member diagnosed with COVID-19 during the pandemic. Many parents (42%) transitioned to remote work during the pandemic, while 21.6% experienced job-loss, and 26.5% experienced reductions in work hours. There were no significant differences across treatment and control groups in any of these COVID-related impacts.

FCU-O engagement. As shown in Table 2, families spent an average of over 2 h on the app, with an average of more than 10 app-visits. Parents recieved an average of 5.45 sessions with coaches, lasting an average of 146.77 min.

**Table 2:** Descriptive statistics for App Usage

	Mean	(SD)	Minimum - Maximum
Total minutes on app	137.05	(56.49)	15.61–352.86
min per module:			
Healthy Behaviors for Stressful Times	30.23	(12.06)	11.28–62.69
Positive Parenting	22.38	(11.44)	4.51–59.10
Rules and Consequences	29.78	(18.60)	6.06–115.90
Support for School Success	12.03	(6.46)	2.00–32.76
Communication	19.21	(11.10)	3.33–60.95
Total visits to app	10.37	(4.34)	1–25
Visits per module:			
Health Behaviors for Stressful Times	3.27	(2.05)	1–12
Positive Parenting	2.68	(1.60)	1–11
Rules and Consequences	2.40	(1.50)	1–9
Support for School Success	1.62	(0.88)	1–5
Communication	2.15	(1.55)	1–10
Number of coaching sessions	5.45	1.73	1–11
Total minutes of coaching sessions beyond initial introduction	146.77	88.92	0–472

Correlational analyses examined associations between app-engagement (number of visits and amount of time) or engagement with coaches (number of sessions, duration of sessions), and demographics or baseline levels of parenting or parent functioning variables. As shown in Supplemental Tables 1, parents who were unemployed or who received financial assistance spent more time on the app, although no variables were significantly correlated with the number of app visits. Parental engagement with coaching sessions (particularly the amount of time in coaching sessions) was associated with a range of demographic factors (including parental unemployment, receipt of financial assistance, and single parenthood), as well as greater baseline levels of parenting difficulties, including greater perceived stress, emotion regulation difficulties, symptoms of anxiety and depression, negative parenting behaviors, and lower levels of positive parenting behaviors and parental monitoring of family routines.

### Intent to Treat Analyses

Descriptive statistics for study outcomes are shown in Table 3, and results for ITT analyses are shown in Table 4. Significant intervention effects were observed for all parental-functioning outcomes (perceived stress, emotion regulation indices, anxiety, and depression), with generally small to moderate effect sizes (although the intervention effect on perceived stress was medium to large). Significant intervention effects were also observed for several parenting domains, including greater positive and proactive parenting (small to medium effects), and reduced negative parenting (medium to large effect). The FCU-O also predicted significant improvements in parental perceptions of family-togetherness, and in co-parenting (both small to medium effects).

**Table 3:** Descriptive statistics for study outcomes

	Control					Intervention			
	T1		T2			T1		T2	
	M	SD	M	SD		M	SD	M	SD
Parent functioning:									
Perceived Stress	1.82	0.51	1.80	0.53		1.83	0.62	1.54	0.60
DERS: Impulse	9.67	3.46	9.43	3.05		9.63	3.77	8.66	3.34
DERS: Strategies	13.19	4.87	13.39	4.41		13.84	5.67	12.62	4.33
Anxiety	2.28	1.65	2.29	1.63		2.36	1.85	1.88	1.57
Depression	8.43	5.34	7.40	4.94		8.53	6.22	6.51	5.23
Parenting:									
Negative parenting	1.17	0.60	1.13	0.58		1.30	0.60	0.93	0.51
Positive parenting	3.94	0.66	3.90	0.74		3.91	0.72	4.13	0.65
Proactive parenting	3.71	0.59	3.68	0.62		3.81	0.66	3.94	0.59
Limit Setting	3.77	0.52	3.81	0.46		3.85	0.50	4.00	0.53
Monitoring family routines	4.50	0.43	4.44	0.48		4.48	0.46	4.54	0.51
Monitoring school activity	4.20	0.60	4.16	0.54		4.29	0.58	4.32	0.59
Family Relations:									
Family togetherness	3.37	0.86	3.32	0.80		3.41	0.89	3.68	0.91
Family conflict	1.82	1.15	1.81	1.13		1.86	1.01	1.52	0.95
Co-parenting	2.99	0.61	2.97	0.72		3.15	0.66	3.28	0.66

**Table 4:** Intervention effects from regression models

	Beta	SE	*p*	Standardized Beta	Effect size
Parent functioning:					
**Perceived Stress**	**− 0.27**	**0.07**	**< 0.001**	**− 0.24**	**0.49**
**DERS: Impulse**	**− 0.80**	**0.39**	**= 0.04**	**− 0.12**	**0.24**
**DERS: Strategies**	**-1.18**	**0.49**	**= 0.02**	**− 0.13**	**0.26**
**Anxiety**	**− 0.45**	**0.21**	**= 0.03**	**− 0.14**	**0.28**
**Depression**	**-1.15**	**0.57**	**= 0.04**	**− 0.12**	**0.24**
Parenting:					
**Negative parenting**	**− 0.25**	**0.06**	**< 0.001**	**− 0.23**	**0.48**
**Positive parenting**	**0.20**	**0.09**	**= 0.04**	**0.14**	**0.28**
**Proactive parenting**	**0.18**	**0.08**	**= 0.03**	**0.14**	**0.28**
Limit Setting	0.14	0.07	= 0.07	0.14	0.23
Monitoring family routines	0.10	0.06	= 0.11	0.10	0.20
Monitoring school activity	0.09	0.07	= 0.20	0.08	0.16
Family relations:					
**Family togetherness**	**0.30**	**0.07**	**= 0.02**	**0.17**	**0.35**
Family conflict	− 0.20	0.14	= 0.15	− 0.09	0.18
**Co-parenting**	**0.16**	**0.07**	**= 0.03**	**0.11**	**0.24**

Note: Effect size d is interpreted as follows: 0.20 = small; 0.50 = medium; 0.80 = large (Cohen, 1988)

## Moderation Analyses

Only one significant interaction between treatment and youth gender was observed, for impulse control problems (beta = 2.05, SE = 0.71, *p* < .01). In follow-up analyses, the intervention effect on parental emotion regulation difficulties was significant for parents of boys (beta = -1.85, SE = 0.51, *p* < .001), but not girls (beta = 0.20, SE = 0.49, *n.s.*). No significant moderation effects by race/ethnicity were observed.

## Discussion

This study examined the short-term impact of a novel adaptation of the FCU-O, designed to support adaptive parenting in response to stressors such as those associated with the COVID-19 pandemic. In particular, a new module, “healthy behaviors for stressful times,” was developed with input from parents and community providers, to support parental self-care as well as youth coping with stress, including stressors due to the pandemic.

Results of the current study showed that the enhanced FCU-O predicted consistent improvements in important aspects of parent functioning at 2-month follow-up. Parents in the FCU-O condition reported significant reductions in perceived stress, improvements in emotion regulation (including impulse control and access to emotion regulation strategies), and reductions in symptoms of anxiety and depression, with effect sizes ranging from small-to-moderate to moderate-to-large in magnitude. Parents also reported significant improvements in crucial parenting skills, including reductions in negative parenting behaviors (e.g. hostile criticism, yelling at child), increases in positive parenting strategies (e.g. use of praise and spending positive time together), and improvements proactive parenting behaviors (e.g. giving reasons for requests, breaking tasks into smaller steps), with effect sizes also ranging from small-to-moderate to moderate-to-large. Improvements in parental perceptions of family-togetherness and the quality of the co-parenting relationship were also observed.

There were, however, several areas of parenting and family relationships for which significant intervention effects were not observed, including parental limit-setting, monitoring, and family conflict. It is possible that these behaviors may take a longer time to change. As we follow families through 4- and 6-month follow-up assessments, we will examine longer-term impacts on these aspects of parenting.

Nevertheless, the overarching pattern of results show that significant improvements in parental mental health, adaptive parenting, and family relationship functioning can be achieved with a relatively brief program incorporating app-based intervention material and coaching support for parents, in order to support effective parenting during stressful times. Parents spent nearly 5 h of intervention time across app-based materials and parent-coaching sessions. While this is relatively brief compared to many parenting interventions, it is comparable to the original school-based trials of the FCU. For instance, in the first large scale RCT of the FCU in with children aged 10–14, families received an average of 8.9 h of in-person intervention, while families received an average of 5.62 h of in-person intervention between grades 7 and 9 in a subsequent school-based trial. This study also builds upon work with the FCU-O prior to the COVID-19 pandemic (Stormshak et al., 2019), and it is worth highlighting that parents in the current trial spent more than twice as much time engaging with the FCU-O’s online material (137.05 min versus 64.98 min in the prior trial). Similarly, parents in the current trial visited the app more than twice as often in the as parents in the prior trial (10.37 visits versus 4.39 visits). The pervasive challenges associated with the COVID-19 pandemic may have led parents to be particularly receptive to a readily accessible parent-focused intervention such as the FCU-O.

In line with this possibility, analyses of factors associated with greater engagement with the FCU-O generally showed that greater parental challenges were associated with greater engagement with elements of the intervention, although results differed somewhat for engagement with app-based materials and with coaching sessions. Parents who were unemployed or reported receiving financial assistance (e.g. unemployment insurance, social security benefits, etc.) generally spent more time engaging with app-based intervention materials. A broader range of baseline factors were associated with engagement with coaching sessions, however. Parental unemployment and receipt of financial assistance were associated with spending more time in coaching sessions, as were single-parenthood and greater baseline levels of parenting difficulties (including greater perceived stress, emotion regulation difficulties, symptoms of anxiety and depression, negative parenting behaviors, and lower levels of positive parenting behaviors and parental monitoring of family routines).

Our results regarding engagement-related factors are in contrast to the broader emerging literature on predictors of engagement with online mental health interventions, which has yielded few consistent predictors of adherence across a range of demographic, treatment-target, or programmatic factors (Beatty & Binnion, 2016). However, these results are generally consistent with research on factors predicting greater engagement with the original in-person version of the FCU. Across trials of the FCU initiated in early childhood, and in early adolescence, greater engagement with the FCU was generally associated with elevations in baseline risk-factors, including conduct problems at school, youth reports of family conflict, parental depression, and the absence of biological fathers from the home (e.g. Connell et al., 2007; Dishion et al., 2014). Overall, the current results add to the literature showing that a relatively brief parent-focused intervention that incorporates app-based intervention materials and a remote-delivery format can be engaging for highly-stressed families, and lead to improvements in critical aspects of parent and family functioning, even during during challenging circumstances such as those associated with the COVID-19 pandemic.

## Limitations and Future Directions

There are several limitations that highlight areas for future research. First, youth functioning was not examined in the current analyses, as youth only completed baseline and 6-month assessments, to minimize youth-burden. Follow-up youth assessments are underway and will be examined in future analyses. Second, although representative of the Pacific Northwest region, the sample was somewhat homogeneous. Future work on the efficacy of the FCU-O with more diverse populations is needed. Third, fathers were far less likely than mothers were to participate. As such, future research is needed to address the extent to which the results for the FCU-O may generalize to fathers. Fourth, power to detect differences across subgroups was was somewhat limited, which may have contributed to finding relatively few significant differences in intervention effects across subgroups. Finally, outcomes were limited to parental self-report measures which could be completed via online surveys. Although this approach is consistent with the parent-centered intervention and the remote-delivery design, the inclusion of independent informants would enhance confidence. Despite limitations, this trial provides promising evidence regarding the effects of FCU-O for supporting family functioning in the face of the stressful effects of the COVID-19 pandemic.
